# Supplementary figures and images for: Study on Liver Sinusoidal Endothelial Cell Fenestrations Based on Cellular Omics-Structure Integration Technology and Its Application in Metabolic Diseases
Source: bioRxiv. 2025 May 19:2025.05.16.653525. Preprint. [Version 1] doi: 10.1101/2025.05.16.653525 (PMC12139957; doi:10.1101/2025.05.16.653525)

Correlation Heatmap for AIZARANI\_LIVER Gene Sets

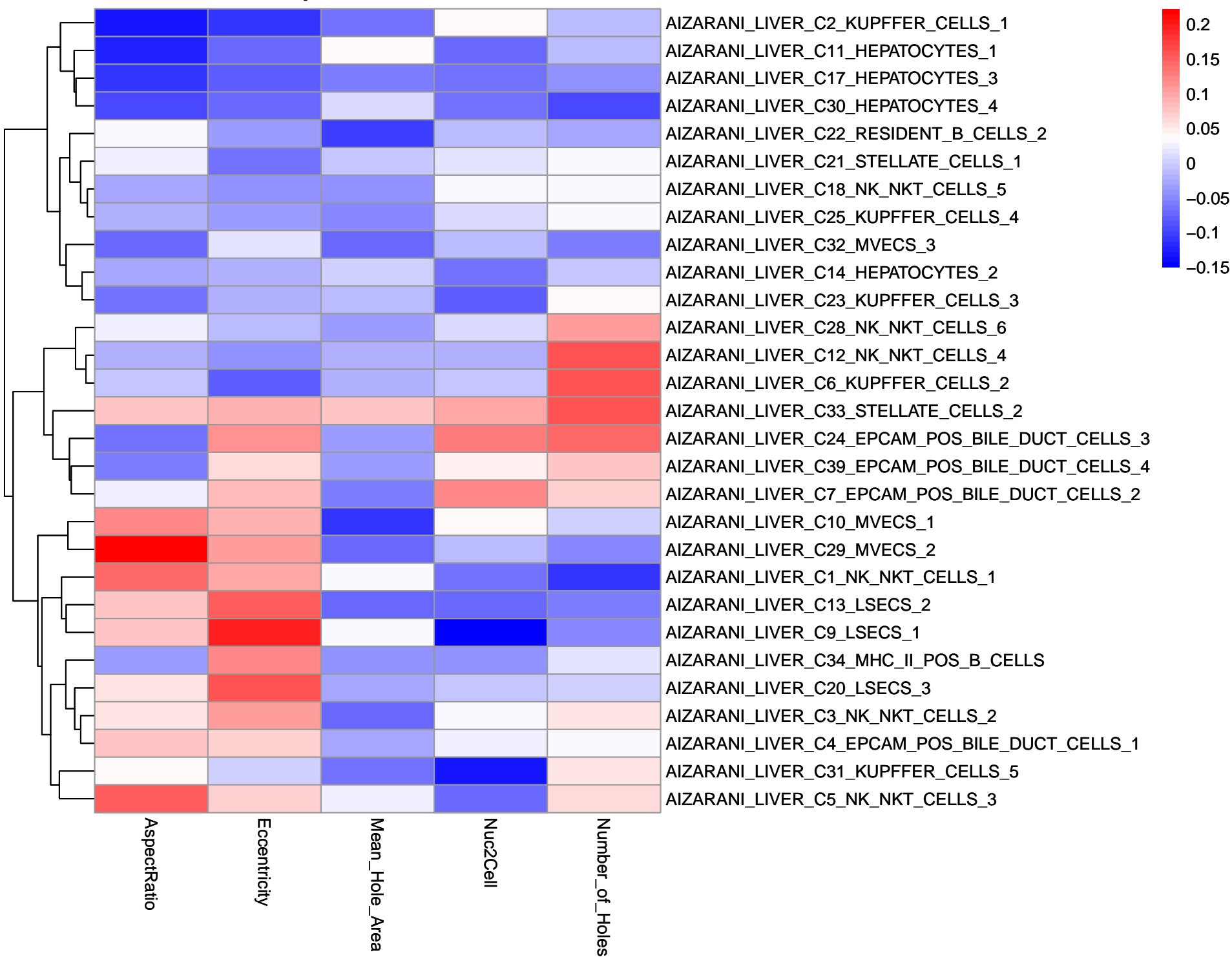

Supplement: Supplement 2 [file media-2.zip › Supplementary files/Supplementary file 39 Correlation_Heatmap_C8_AIZARANI_LIVER_Gene_Sets.pdf]

Correlation Heatmap for KIDNEY Gene Sets

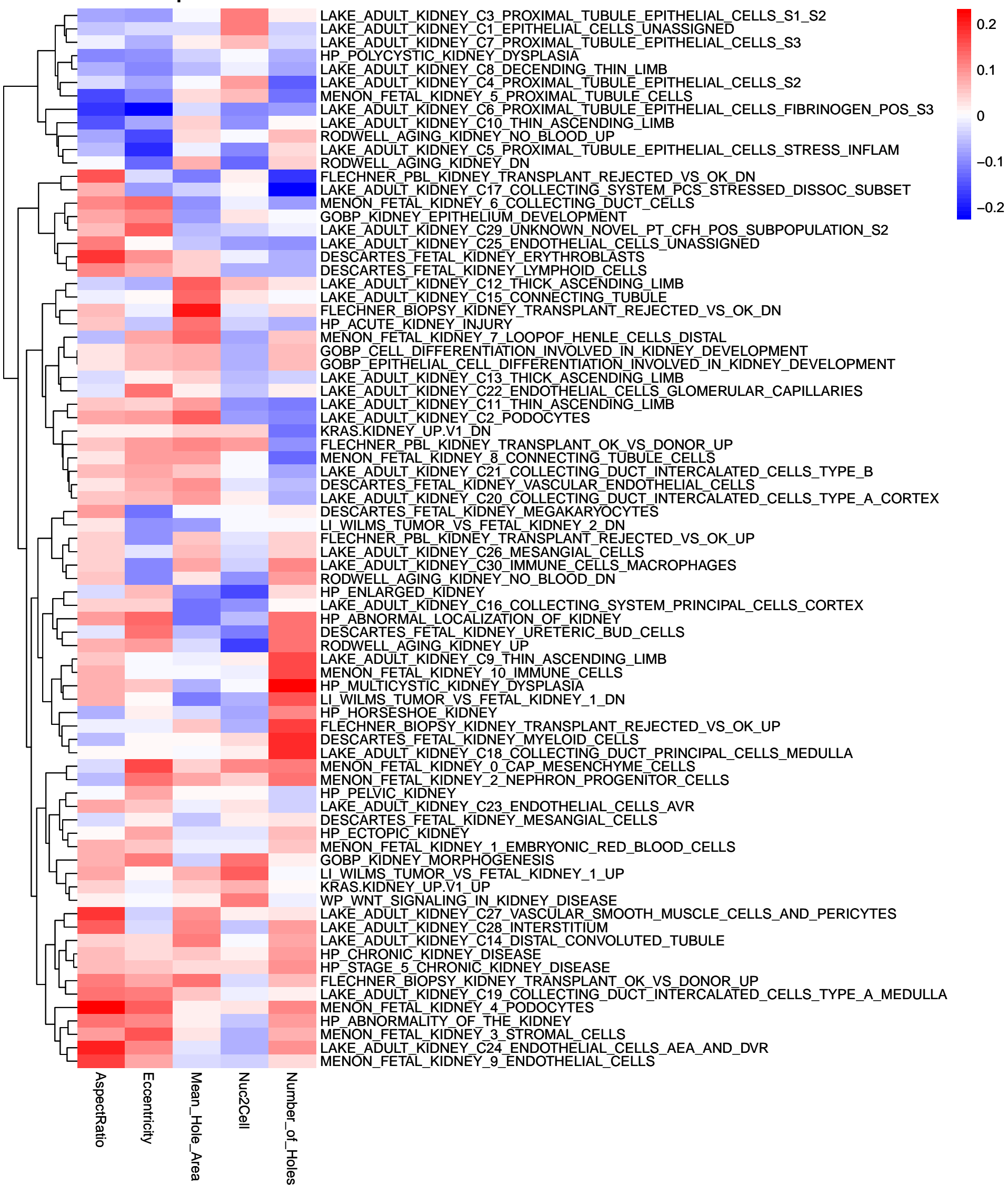

Supplement: Supplement 2 [file media-2.zip › Supplementary files/Supplementary file 38 Correlation_Heatmap_KIDNEY_Gene_Sets.pdf]

Volcano Plot for Gene Sets  
Comparing Mean\_Hole\_Area and Number\_of\_Holes

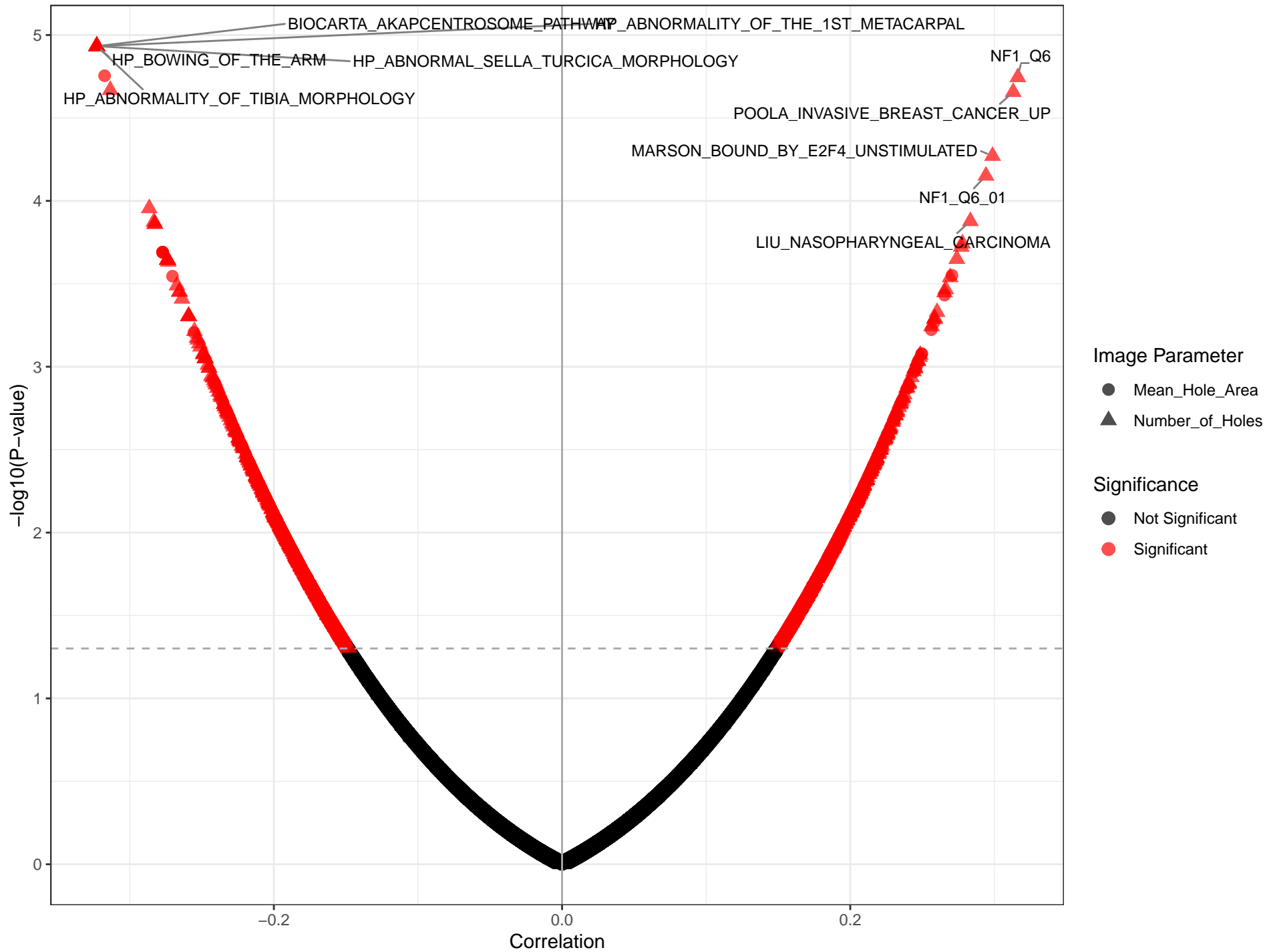

Supplement: Supplement 2 [file media-2.zip › Supplementary files/Supplementary file 29 Gene_Sets_volcano_plot.pdf]

Correlation Heatmap for H Gene Sets

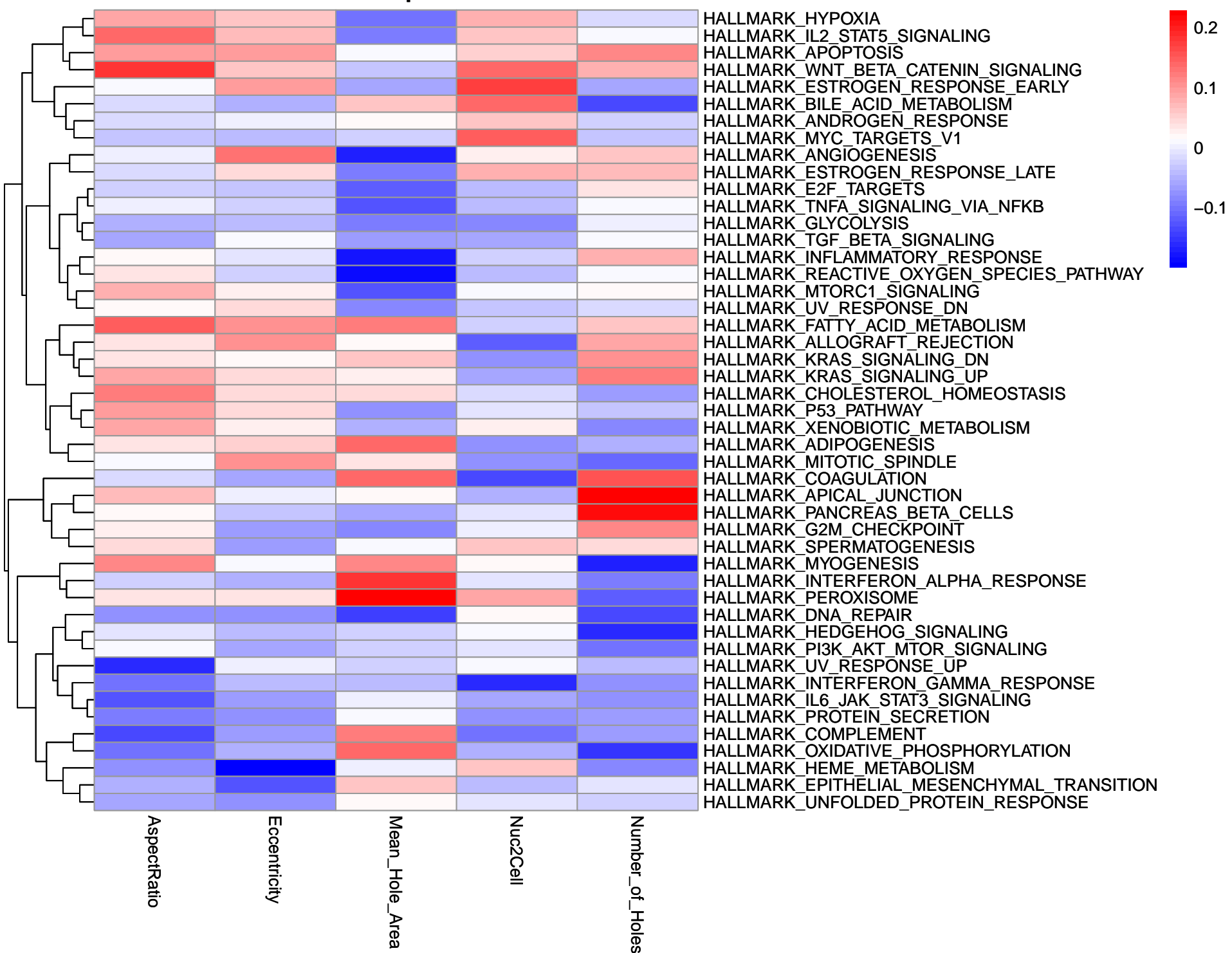

Supplement: Supplement 2 [file media-2.zip › Supplementary files/Supplementary file 37 Correlation_Heatmap_Hallmark_Gene_Sets.pdf]

# Volcano Plot for Gene Sets

Number\_of\_Holes

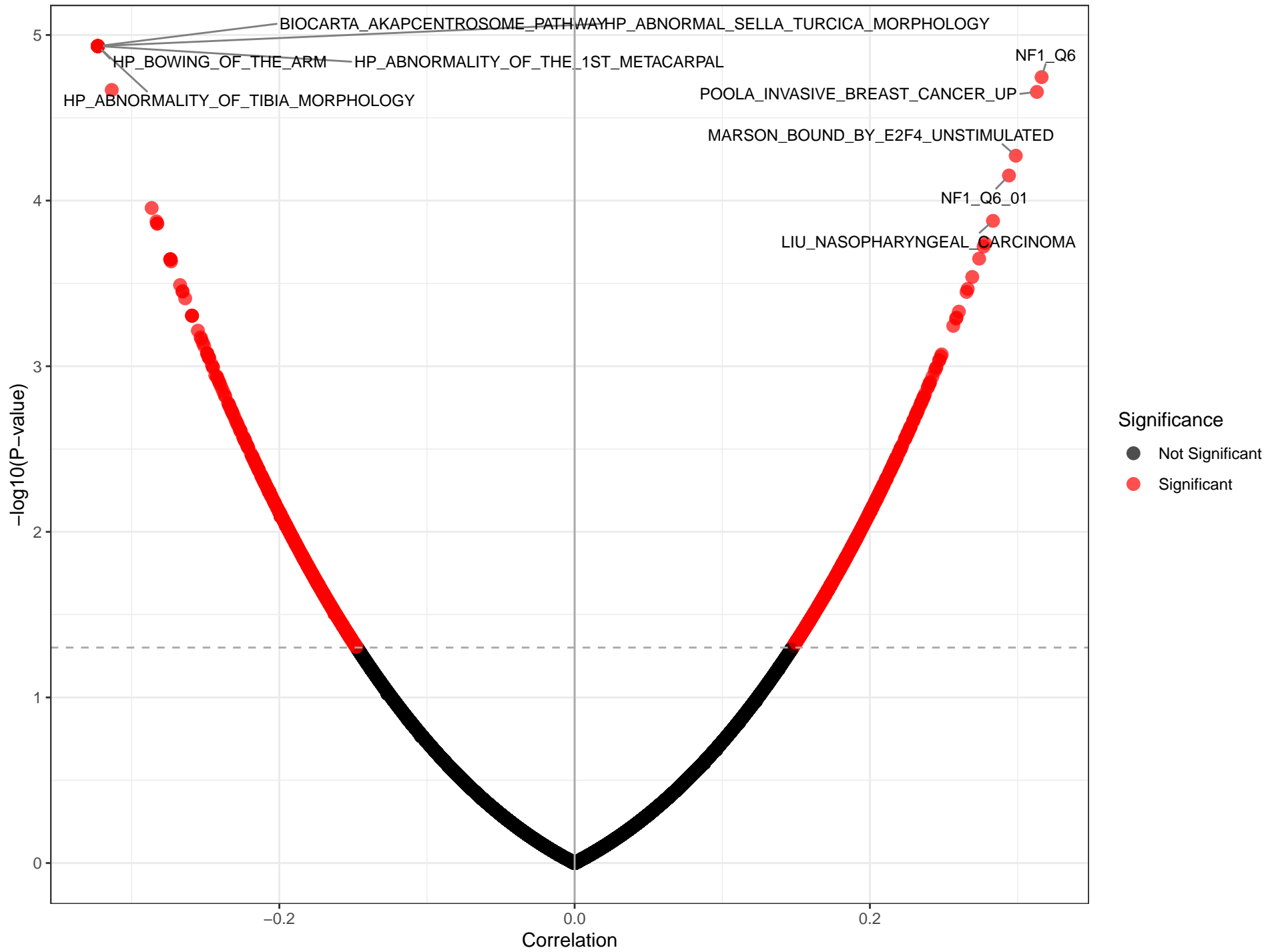

Supplement: Supplement 2 [file media-2.zip › Supplementary files/Supplementary file 31 Number_of_Holes_volcano.pdf]

# Volcano Plot for Gene Sets

Mean\_Hole\_Area

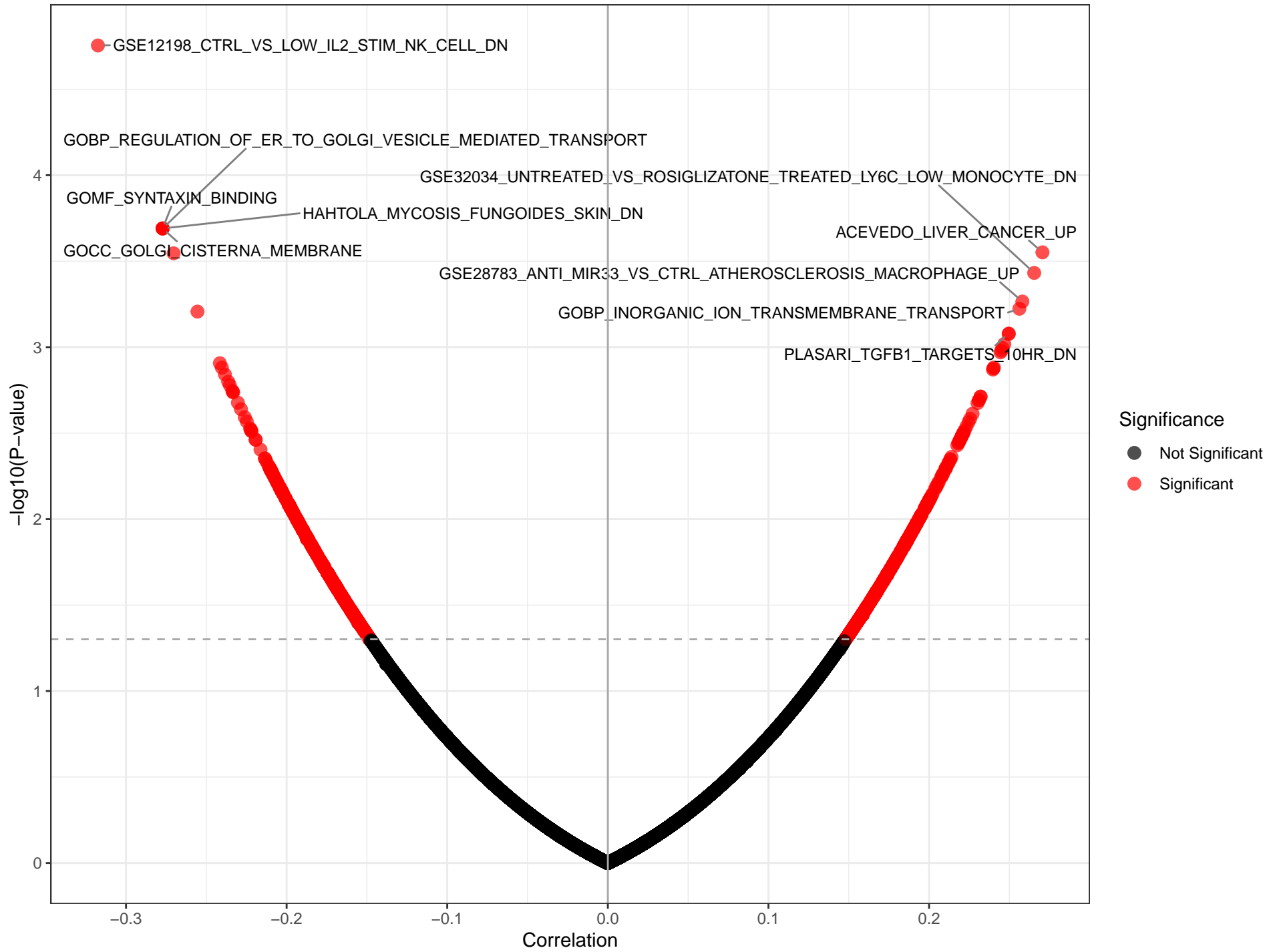

Supplement: Supplement 2 [file media-2.zip › Supplementary files/Supplementary file 30 Mean_Hole_Area_volcano.pdf]
